# Microstructure and Mechanical Properties Analysis of Al/Cu Dissimilar Alloys Joining by Using Conventional and Bobbin Tool Friction Stir Welding

**DOI:** 10.3390/ma15155159

**Published:** 2022-07-25

**Authors:** Kishan Fuse, Vishvesh Badheka, Ankit D. Oza, Chander Prakash, Dharam Buddhi, Saurav Dixit, N. I. Vatin

**Affiliations:** 1 Department of Mechanical Engineering, School of Technology, Pandit Deendayal Energy University, Raysan, Gandhinagar 382007, India; kishan.fuse@sot.pdpu.ac.in (K.F.); vishvesh.badheka@spt.pdpu.ac.in (V.B.); 2 Department of Computer Sciences and Engineering, Institute of Advanced Research, Gandhinagar 382426, India; ankit.oza@iar.ac.in; 3 School of Mechanical Engineering, Lovely Professional University, Phagwara 144411, India; 4 Division of Research and Development, Lovely Professional University, Phagwara 144411, India; 5 Peter the Great St. Petersburg Polytechnic University, 195251 Saint Petersburg, Russia; dbuddhi@gmail.com (D.B.); vatin@mail.ru (N.I.V.); 6 Division of Research & Innovation, Uttaranchal University, Dehradun 248007, India

**Keywords:** bobbin tool, friction stir welding, dissimilar, intermetallic, microhardness, microstructure

## Abstract

The feasibility of producing welding joints between 6061-T6 aluminum and pure copper sheets of 6 mm thickness by conventional friction stir welding (CFSW) and bobbin tool friction stir welding (BTFSW) by using a slot-groove configuration at the joining surface was investigated. The microstructure of the welded samples was examined by using an optical microscope and X-ray diffraction. Furthermore, the mechanical properties of the weld samples are compared based on the results of the tensile test, hardness measurement, and fractography test. The slot-groove configuration resulted in the presence of a bulk-sized Al block on the Cu side. The microscopic observations revealed the dispersion of fine Cu particles in the stir zone. The presence of intermetallic compounds (IMCs) CuAl_2_, which are hard and brittle, lowered the strength of the weld joints. The strength of the weld joints produced with BTFSW was superior to that of the C-FSW. The maximum hardness values of 214 HV and 211 HV are reported at the stir zone for BTFSW and CFSW, respectively. The fracture location of all the joints was at the intersection of the stir zone and the thermomechanically affected zone was on the Cu side.

## 1. Introduction

The dissimilar material joining in one of the recent challenges faced by manufacturing industries. The amalgamation of Al and Cu is of extreme interest in electricity, cryogenic and refrigeration fields pertaining to benefits such as weight reduction and cost saving [1,2]. This necessitates a reliable joining technique for fabricating Al/Cu components. The challenges in joining of Al/Cu combination is due to large dissimilarities in their physical, thermal, and chemical properties, such as differences in melting temperatures and the strong affinity between Al and Cu [3]. The formation of brittle intermetallic compounds (IMCs) produces joints with less mechanical strength. Thus, the conventional fusion welding techniques are not fit for such joining as it requires melting of material followed by cooling. 

Solid-state welding has emerged as a solution for joining dissimilar material systems. One novel type of solid-state welding is conventional friction stir welding (CFSW). The process can join Al and Cu at low heat input as it utilizes friction heat and mechanical deformation to produce joints [4,5,6]. The low heat input suppresses the formation of excessive IMCs and results in better joint characteristics [7]. Comprehensive research has been reported to investigate and improve the joint strength of Al/Cu joining by using the conventional FSW technique. 

Several process parameters affect the weld joint quality during CFSW of Al/Cu joining. Shankar et al. [8] investigated the effect of welding speed and tool offset on FSW of dissimilar Al 1050 and oxygen-free copper joining. They reported the highest joint strength, 91% AA 1050 alloy strength. Khajeh et al. [9] pointed out that welding speed (W) and traverse speed (V) ratio (W/V) significantly affect joint properties as they stand for heat input. They reported IMCs and voids as prime reasons for lower strength and ductility in non-optimum joints. Hou et al. [10] investigated tool offset during FSW of 6061 aluminium alloy and commercially pure copper via FSW. They reported an increase in tensile strength with an increase in tool offset from 0 mm to 1.4 mm. They also found peak temperature dependency on tool offset. Zhang et al. [11] produced a sound weld between 1060 aluminium alloy and annealed a pure copper sheet by using CFSW at a tool rotating speed of 1050 rpm and a weld travel speed of 30 mm/min. They inferred metallurgical bonding between base materials to form an intercalation structured at the intersection of Cu and weld nugget area. The hardness increment was mainly due to higher dislocation density and developed dislocation loops. 

Optimization of process parameters is essential to recognize the best process parameter window during CFSW of Al/Cu joining. Mahdianikhotbesara et al. [12] optimized micro FSW for producing dissimilar material joining between 1050 aluminium and pure copper alloys. They performed experiments by using Taguchi L9 arrays. They found the rotation speed of the tool and welding speed as the most significant parameters affecting tensile strength. Eslami et al. [13] adopted the Taguchi technique for experimental design to optimize process variables during FSW of aluminum 1050A and copper EN CW004A. They concluded that the lowest offset of 1.4 mm led to the best electrical and mechanical properties. Sahu et al. [14] presented the fuzzy-grey Taguchi technique for optimizing Al/Cu dissimilar FSWed joints. They varied rotational tool speed, traverse speed, plunge depth, and offset distance of tool. They measured the effect of selected parameters in terms of compressive strength, percentage of elongation, angle of bend, weld bead thickness, and average hardness at the stir zone (SZ). 

Enhancing joint properties by using different welding strategies remained an active area of research in CFSW of Al/Cu joining. Sahu et al. [15] conducted CFSW of Al/Cu with Ti, Ni, and Zn foil as an interlayer to enhance joint properties. They reported enhanced metallurgical and mechanical characteristics with Zn interlayer due to the formation of uniform, thin, and continuous IMCs. Hou et al. [16] cold sprayed the Cu plate with a Ni coating of 90 µm and further FSWed to a 6061 aluminium alloy in a butt configuration. They found significant improvement in mechanical strength. They reported average peak stress and percentage elongation of 190 MPa and 14%, respectively. Argesi et al. [17] used SiC nano-composites to decline the adverse effect of IMCs during FSW of pure copper and Al 5754 alloy. They observed a reduction in Al and Cu grain size in the SZ due to SiC particles. They also found a significant increase in microhardness of the weld zone from 160 HV to 320 HV. Zhang et al. [18] replaced the surface-to-surface joint configuration at the joining area with a tooth-shaped joint configuration for bonding Al/Cu dissimilar metals by using CFSW. This proved a better design to tailor the base materials content into the weld. They reported a failure load of 9.6 KN and 7.9 KN with tool-shaped joint configuration and routine butt configuration, respectively. 

Bobbin tool friction stir welding (BTFSW) is a recently developed variant of the CFSW technique. The exception in BTFSW is that the tool of BTFSW is attached with an additional shoulder at the lower side of the pin, known as the lower shoulder [19,20]. The lower shoulder helps avoid use of a backing plate, which is an integral part of CFSW. The BTFSW offers many advantages over CFSW, such as eliminating root flaw defects like lack of penetration, full penetration, simplified fixture, uniform through-thickness grain structure, effective utilization of generated frictional heat etc. [21,22,23]. The investigation using BTFSW includes studying the effect of rotation speed [24,25], welding speed [26,27], optimization of process parameters [28], comparative study of CFSW and BTFSW [29], and numerical modelling [30,31]. 

The literature study revealed that conventional FSW had been extensively investigated for joining of dissimilar Al/Cu materials considering welding process variables such as tool rotating speed, tool traverse speed, axial force, tool pin offset, positioning of base plates, the effect of geometrical features of the tool such as tool diameter, pin diameter, pin length etc. However, the current research needs more attention on the other welding strategies, such as the alignment of base plates at the joining surface (slot-groove configuration). On the other hand, despite the proven potential of BTFSW over CFSW, limited research is present in the literature. The research on BTFSW was focused on similar material joining only. The use of BTFSW for dissimilar Al/Cu joining needs to be addressed to explore the technique. Thus, the present work addresses the research questions about joining Al/Cu dissimilar materials by using conventional FSW and BTFSW. The slot-groove configuration fixes the base plates at the joining surface. Weld samples of Al/Cu joints will be put through mechanical and microstructural characterization to ensure their quality.

## 2. Experimental Methodology

**Figure 1:**
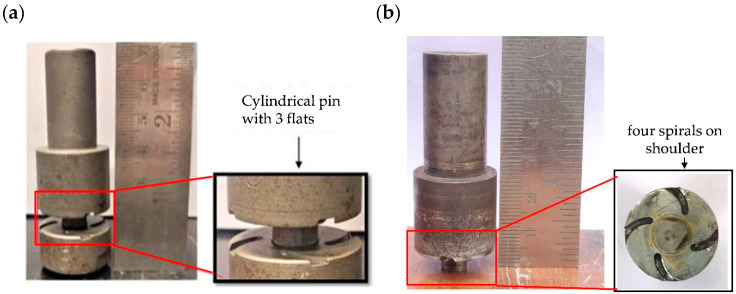
Stirring tool used for experiments (**a**) BTFSW tool (**b**) CFSW tool.

This study aimed to make dissimilar material joining by using slot-groove joint configuration by using BTFSW and further compared with the CFSW joint. The base plates used for the study were 6061-T6 aluminium and pure copper. The chemical composition (% weight) of the Al plate is Si-0.457%, Fe-0.552%, Zn-0.010%, Cu-0.173%, Mg-0.937%, Mn-0.106%, and Al-balanced. The copper plate used contained 99.9% Cu. The schematic of the joint configuration and experimental setup is shown in Figure 1. During the experiment, copper was placed on the advancing side (AS), and 6061-T6 aluminium was placed on the retreating side (RS) for proper material mixing. In this work, both the plates used were 65 mm × 65 mm × 6 mm dimensions. The butt joint configuration is attempted in this study.

**Figure 2:**
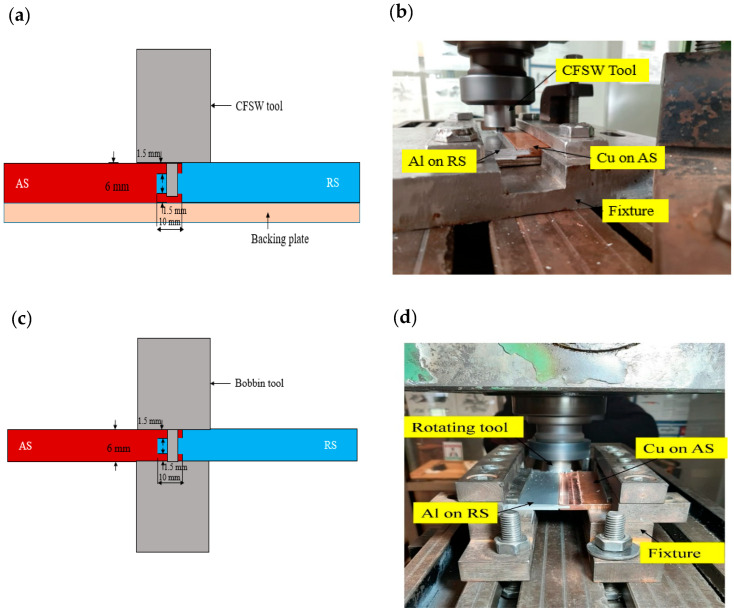
Joint configuration for dissimilar Al/Cu joining using CFSW. (**a**) Schematic (**b**) experimental set-up and BTFSW. (**c**) Schematic (**d**) experimental set-up.

The welding experiments were worked on the vertical milling machine. The tool rotation speed of 1500 rpm and travel speed of 31.5 mm/min was used in both the welding conditions. The tilt angle of 2° was used in CFSW. The H13 tool steel was used to make the non-consumable tools for CFSW and BTFSW. The BTFSW tool design comprised of top and bottom shoulder having 24-mm diameter. The shoulders were provided with four spiral grooves each, which assisted in flowing the material from the outer shoulder edges to the tool’s pin. The cylindrically shaped tool pin of 8-mm diameter was designed with a three flats feature. The length of the tool pin was 6 mm. The CFSW tool used during experiments had all the dimensions and features similar to the BTFSW tool except for the lower shoulder. The Figure 1 shows the CFSW and BTFSW tools used for the experimentation. The edge of the joining plates was modified to ensure proper mixing of material from AS to RS. The slot-groove configuration was prepared in such that copper plate had a groove which worked as the female part, and aluminium had a slot which worked as the male part. A longitudinal groove of size 130 mm × 10 mm × 3 mm was prepared (as a female part) along the thickness of the copper plate. Further, the male counterpart in 6061-T6 aluminium was machined to a thickness of 3.5 mm and width of 10 mm. It was inserted into the female copper groove as a press-fit before welding, as shown in Figure 2. 

After dissimilar Al/Cu welding, the weld samples were sectioned transversely to the welding route. The cut samples were prepared for the metallographic study by grinding, polishing, and further etching. The modified Keller’s reagent was used on the Al side, and the Cu side was etched with 5 mL FeCl_3_, 10 mL ethanol, 10 mL HCl and remaining distilled water. Metallographic inspections were carried out by Olympus-GX51 optical microscopy (Shinjuku, Japan). The Vickers microhardness indentation machine (NEXUS 4302) was used to assess microhardness. It is measured along the centerline of polished cross-sections at 1-mm intervals. During the measurement, a load of 300 g was used with a dwell time of 10 s. The tensile testing was executed at ambient temperature conditions by using computer-controlled UTM (FSA/M-100) with a speed of 1 mm/min. X-ray diffraction (XRD) studies can be used to confirm the production of distinct IMCs at the NZ.

## 3. Results and Discussion

### 3.1. Surface Appearance of the Joints

**Figure 3:**
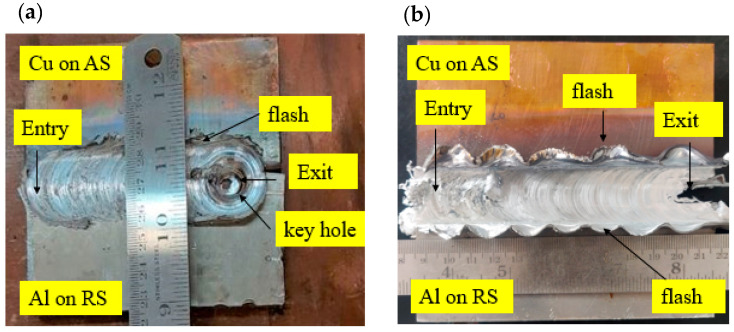
The top surface images of the Al/Cu joints welded with (**a**) CFSW, (**b**) BTFSW.

Figure 3 presents morphologies of the top surface of Al/Cu butt joints welded under CFSW and BTFSW conditions. It can be observed that the weld surface is present with uniform ripples without any surface defects such as cracks or tunnels. The CFSW joint is present with a keyhole at the exit, whereas the BTFSW tool comes out of the joining zone by cutting the plates at the exit. The flash can be observed only on AS in the CFSW joint, whereas the flash is present on both sides in the BTFSW joint. The flash in CFSW is mainly comprised of aluminium material. The specific movement of plasticized material during FSW instigated the formation of flash. In the present work, 6061-T6 aluminium is less viscous compared to copper. Hence, during the stirring process, less-viscous aluminium smoothly flowed from the advancing side to the retreating side, resulting in a flash. The flash on both sides in BTFSW can be attributed to more plunge depth at the top surface. In BTFSW, plunge is decided from pin length. When pin length in BTFSW is less than the thickness of the base plates, the shoulders penetrate more on either top or bottom surface. In the present work, it can be concluded that more penetration of the upper shoulder of the BTFSW tool on the top surface of the work material resulted in a flash on both sides. 

### 3.2. Macrostructure Observations

**Figure 4:**
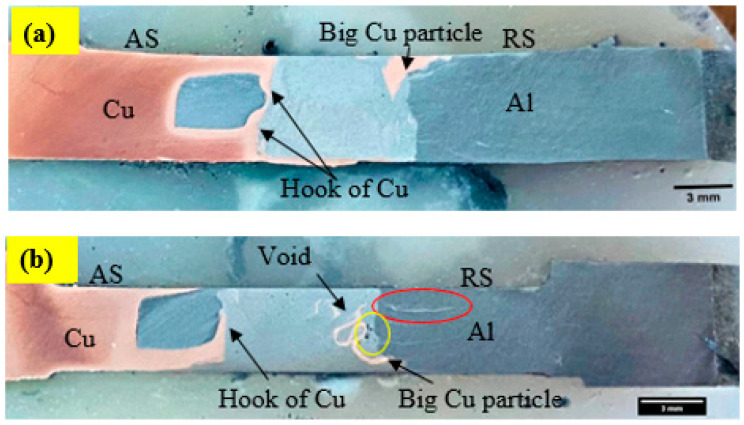
Macrostructure of Al/Cu joint by (**a**) CFSW (**b**) BTFSW.

The macrostructure observed for dissimilar Al/Cu joints using CFSW and BTFSW is shown in Figure 4. It can be observed that the macrostructure for the CFSW is similar to BTFSW. The various sizes and shapes of the Cu particles can be seen distributed in a wide area of the joints. The hook formation phenomenon can also be observed in both joints. The SZ exhibits a heterogeneous structure. The interface of Al and Cu can be identified as unsmooth, which can be reasoned to scratching copper particles from the copper base material with the rotating tool. The big Cu particle can be seen at the intersection of SZ and the thermomechanically affected zone (TMAZ) on RS. The big Cu particle in CFSW is located at the top surface while in the mid and bottom of SZ for BTFSW. This can be attributed to the single shoulder in CFSW and the double shoulder arrangement in BTFSW. Figure 4b reveals that the Al has formed a bonding with Cu in the form of a hook. The hook formation can be attributed to a special slot-groove joint configuration. It is observed that some big-sized copper particles have dispersed at the intersection of SZ and TMAZ on RS. The stir zone was observed to be defect free. The red circle shows the fine copper particles dispersed at the top surface in the aluminium. Voids can be seen at the intersection of SZ and TMAZ on RS, as shown by the yellow circle. One of the reasons for forming the void is the presence of big Cu particles in the zone, which restricts the material flow in the region. 

### 3.3. Microstructure Observations

**Figure 5:**
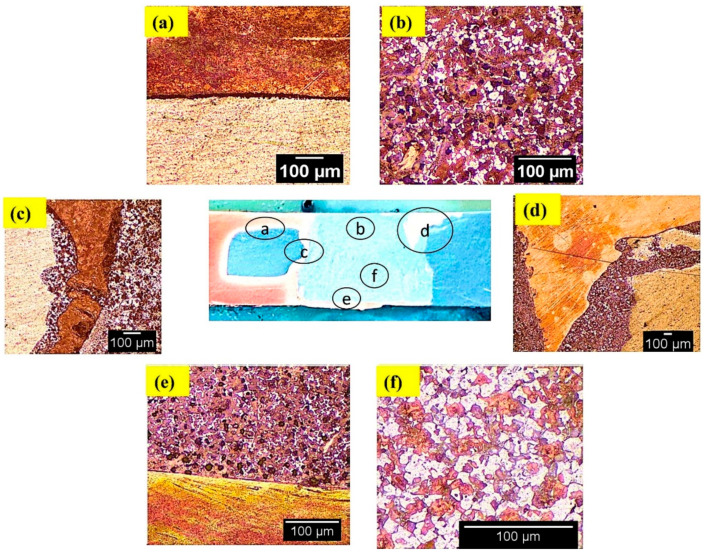
Microstructure in Al/Cu joint formed by CFSW at (**a**,**b**,**d**) top, (**c**) middle, (**e**,**f**) bottom.

**Figure 6:**
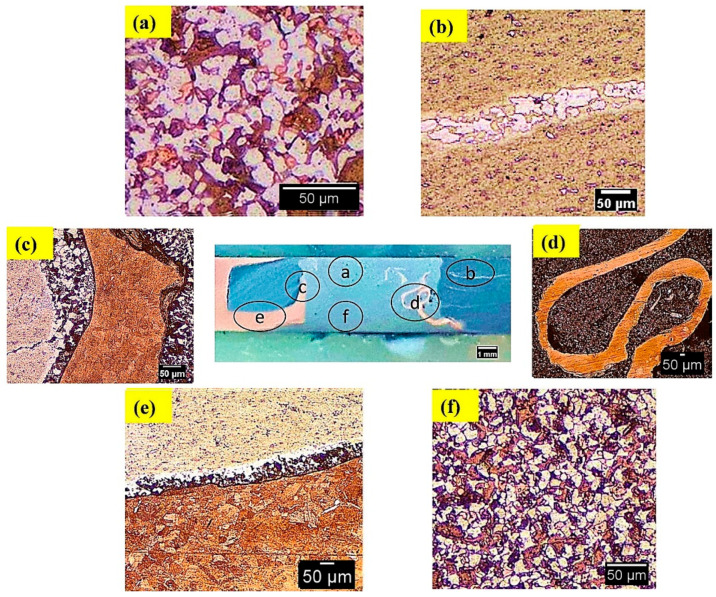
Microstructure in Al/Cu joint formed by BTFSW at (**a**,**b**) top, (**c**,**d**) middle, (**e**,**f**) bottom.

Microstructural features of the welds produced under CFSW and BTFSW are shown in Figure 5 and Figure 6, respectively. The SZ in CFSW presents excellent bonding of Al and Cu, as shown in Figure 5a. No defect was observed in SZ. The SZ can be identified with the number of Cu particles in the Al matrix, which is obviously different from similar welding material. More dense dispersion of fine Cu particles was observed at the top than at the bottom, as shown in Figure 5b,f. It can be attributed to the combined effect of the pin and shoulder in the dispersion of Cu at the top surface. However, dispersion at the bottom was only due to pin effect. No bonding between the Al and Cu can be seen at some locations, as shown in Figure 5a. The hook intersection is surrounded by a small amount of Al/Cu mixed material on one side than the other (Figure 5c). The big Cu particle can be seen at the intersection of SZ and TMAZ on RS in CFSW, as shown in Figure 5d. The strong mechanical stirring produced by the rotating tool results in the scratching of Cu particles from the Cu base and further deposited in the aluminium matrix on RS. 

Figure 6a shows how dissimilar joints produced by BTFSW revealed that the SZ consists of a composite structure of Al and Cu materials. In SZ, superior bonding between Al and Cu was highlighted. The size of Cu particles is larger in SZ at the top compared to SZ at the bottom as shown in Figure 6a,f. This can be attributed to more net heat at the bottom surface than at the top surface in BTFSW. The net heat is the difference between the heat generated and heat loss at respective locations. In presented study, during BTFSW, the friction heat generated is the same at top and bottom surfaces due to the same size of the shoulder diameters. However, the amount of heat loss is more at top than on the bottom side. This is due to heat dissipation take place at the top surface via heat conduction into spindle and fixture and heat convention into the air. However, in the case of the bottom shoulder, only heat convention into the air is the mode of the heat dissipation, thus resulting in more net heat at the bottom side. Thus, more net heat at bottom might have caused the copper to stir more intensely and resulted in the higher deformation, reasoned in the dispersion of smaller copper particles in the Al matrix at bottom of the SZ. The dispersion of Cu base material particles in the Al matrix at RS can be observed in Figure 6b. This may be due to severe stirring of the base material and movement of Cu from AS to RS. The hook of Cu is surrounded by excellently boned Al and Cu, as seen in Figure 6c. The interface mixing of Al and Cu at the bottom is presented in Figure 6e.

### 3.4. Tensile Properties

**Figure 7:**
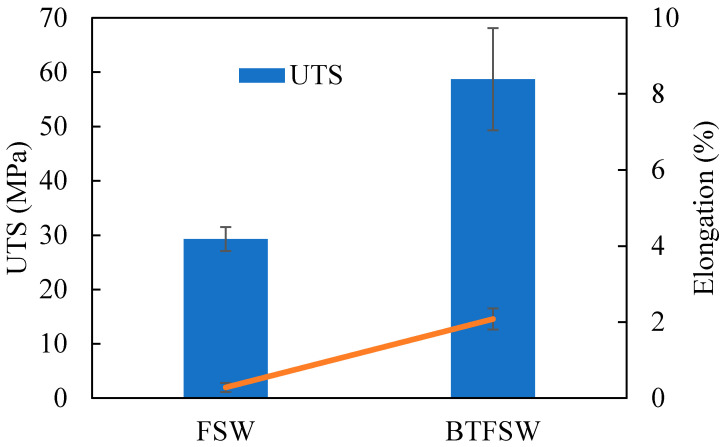
Tensile properties of dissimilar Al/Cu joint produced by FSW and BTFSW.

The UTS and fracture to elongation for the welds produced by CFSW and BTFSW are shown in Figure 7. The UTS of the welds produced under CFSW and BTFSW is reported as 29.29 ± 2.19 MPa and 58.70 ± 9.41 MPa, respectively. The lower UTS for CFSW can be attributed to less strength at hook intersections. The hook intersection acted as a crack initiation location in CFSW. On the other hand, the hook of Cu formed in the BTFSW joint is surrounded by intermixed Al and Cu on both sides. It restricted easy crack formation at hook location resulting in better strength than CFSW. However, the achieved strength in both the welds is not acceptable to the strength of base materials.

The low strength in both the joints may be the presence of intermetallic (IMCs). It is known that the IMC layer is the most important for metallurgical bonding between Al and Cu at the interface. But the excessive thickness of the IMC layer is drastically detrimental to the joint properties [3]. Cu particle-mixing in the Al matrix is typically problematic due to incompatibilities in the chemical and physical properties of the joining metals, resulting in the development of IMCs. The enormous number of IMCs has an unfavourable impact on the weld joint strength and hardness as IMCs are hard and brittle. In CFSW and BTFSW joints, the end of extruded part of Al has not jointed properly in the slot of Cu, as presented in Figure 5a and Figure 6e. The joining between the base materials was due to a formed Cu hook during the process, which resulted in reported strength in the joint. The fracture to elongation is observed as very low as 2.08% and 0.28% for the welds produced by BTFSW and CFSW, respectively. Low elongation is caused by the presence of a large number of hard and brittle IMCs as well as void defects.

### 3.5. Microhardness Observations

**Figure 8:**
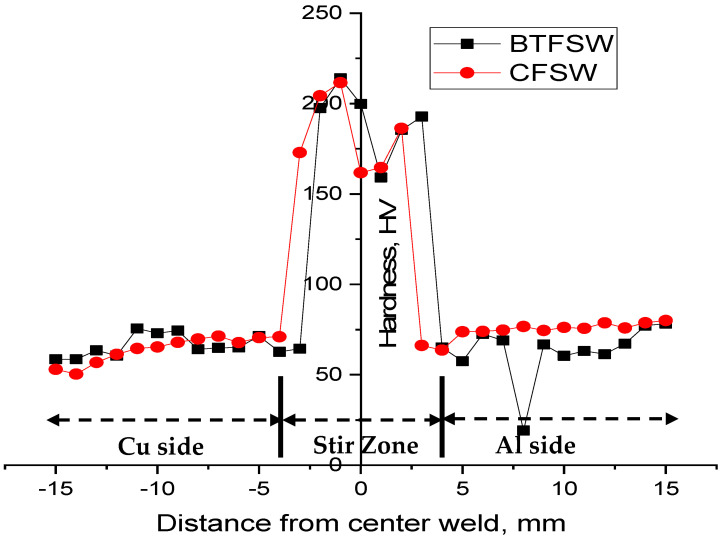
Microhardness of dissimilar Al/Cu joint produced by FSW and BTFSW.

Figure 8 shows the microhardness distribution of the joints produced by CFSW and BTFSW. The peak hardness at the SZ for both the welds can be seen in Figure 7. Maximum hardness values of 211 HV and 214 HV are reported at the stir zone for CFSW and BTFSW, respectively. The hard IMCs presence and fine-grain microstructure are attributed to the higher hardness value in SZ compared to base materials. The fine grains are induced by dynamic recrystallization caused by the combined role of heat and stirring force. The presence of fine grains and extremely thin IMCs layer in the Cu side share area resulted in marginally higher hardness (See Figure 5 and Figure 6). Lower hardness was recorded at the Al side shear zone. The substantial hardness difference between Cu and Al sides in the weld nugget zone (WNZ) is not observed for both samples. This is due to slot and groove configuration instead of regular face-to-face butt configuration because in the slot and groove configuration, the copper is only in contact with aluminium from top and bottom.

### 3.6. XRD Analysis

**Figure 9:**
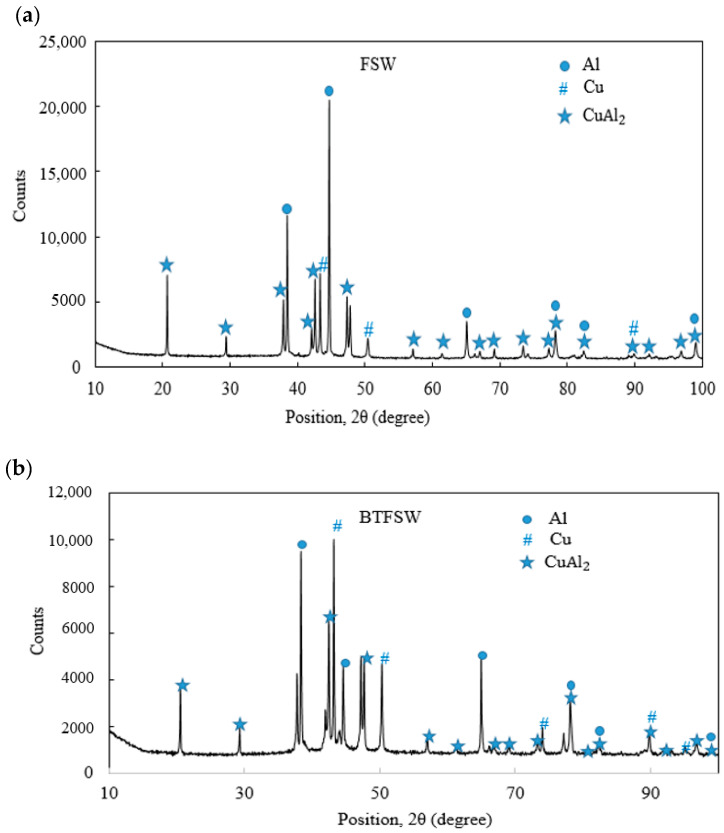
XRD results (**a**) CFSW (**b**) BTFSW.

XRD analysis was performed on the typical dissimilar FSW and BTFSW Al/Cu joints cross-section to reveal the existent phases, especially IMCs. The results of the XRD analysis are presented in Figure 9. The Al–Cu binary equilibrium phase diagram indicates some of the commonly found Al−Cu IMCs as Al_3_Cu_4_ (ζ2), Al_4_Cu_9_ (γ2), Al_2_Cu_3_ (δ), AlCu (η2) and Al_2_Cu (θ). It is reported that the Al_2_Cu (Al-rich phase) and Al_4_Cu_9_ (Cu-rich phase) are the initially formed IMCs near the Al side and Cu side, respectively. In this study, only Al_2_Cu were found in both samples. However, other compounds, such as AlCu, Al_3_Cu_4_, and Al_2_Cu_3_, should also be formed per the Al–Cu phase diagram. Despite the tool pin’s high stirring activity, stronger CuAl_2_ peaks in the SZ suggest an insufficient contact time [32]. The formation of CuAl_2_ IMCs is mainly due to small Cu particles [33].

### 3.7. Fractography

Table 1 presents fracture features of the welds produced by BTFSW and CFSW. All the joints failed at the intersection of TMAZ and SZ on the Cu side. The flat fracture surfaces of all the joints indicate failure is predominantly by brittle fracture. As load increases in the tension test, the tiny cavities in SZ expand rapidly and becomes cracks from which the sample failure starts. Also, inhomogeneous structure in the weld zone may have affected the failure behaviour of the samples [12]. Higher heat input must have promoted the formation of lamellar structures with substantial IMCs, which would harm the samples mechanical properties. 

## 4. Scope for Future Work

The research and development work in dissimilar materials joining by using CFSW has been studied to a large extent with a wealth of process knowledge. But, BTFSW which is an emerging daughter technology of CFSW has been investigated to a lesser extent in joining dissimilar materials even though earlier research on BTFSW showed remarkable advantages of it over CFSW. There is a clear need to understand the feasibility of the BTFSW in the dissimilar material joining. The effect of process parameters such as rotation speed and welding speed can be researched in 6061-T6 aluminium-pure copper joining by using BTFSW. Furthermore, the slot-groove configuration can also been researched by changing male and female combination to know the effect of it. The research can be extended to know the effect of placement of joining materials on AS and RS. The optimization of process parameters by using optimization techniques, which gives correlative model between input parameters and the desired quality output, needs to be explored. 

## 5. Conclusions

This study applied the novel implementation of the bobbin tool technique for joining dissimilar 6061-T6 aluminium and pure copper. The slot-groove configuration’s feasibility for joining dissimilar materials was also attempted. Furthermore, the Al/Cu dissimilar welded joints by using the BTFSW technique were compared with conventional FSW. The concluding remarks of this study can be summarized as follows.

- The research on dissimilar Al/Cu joining by using slot-groove configuration demonstrated that some of the fine copper particles dispersed at the top surface of the aluminium. The voids were present at the intersection of SZ and TMAZ on RS for both the joints.
- Maximum hardness values of 214 HV and 211 HV were recorded at the stir zone for BTFSW and CFSW, respectively. The increased hardness value in SZ against the base metals was credited to the newly formed hard IMCs and fine-grain microstructure.
- XRD analysis of both the samples revealed the presence of Cu2Al IMC in the stir zone of CFSW and BTFSW joints.
- All specimens had a brittle fracture, and the specimens fractured mostly at the intersection of SZ and TMAZ on the Cu side.
- The results of the presented investigation will help explore the wide advantages of the bobbin tool technique in joining dissimilar materials.

## Figures and Tables

**Table 1:** Fracture features of the tensile specimens under BTFSW and CFSW.

Welding	Fracture Sorphologies	Fracture Location	Remarks
BTFSW	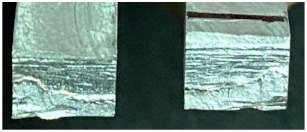	The intersection of SZ and TMAZ on the Cu side	Brittle fracture
	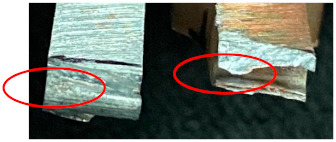	The intersection of SZ and TMAZ on the Cu side	Fracture initiated from the end of extruded Al
CFSW	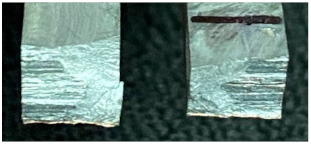	The intersection of SZ and TMAZ on the Cu side	Brittle fracture
	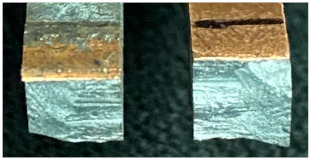	The intersection of SZ and TMAZ on the Cu side	Brittle fracture

## Data Availability

Not applicable.
